# Using Belgian pharmacy dispensing data to assess antibiotic use for children in ambulatory care

**DOI:** 10.1186/s12887-021-03047-7

**Published:** 2022-01-03

**Authors:** Hannelore Dillen, Ruben Burvenich, Tine De Burghgraeve, Jan Y. Verbakel

**Affiliations:** 1 EPI-Centre, Department of Public Health and Primary Care, KU Leuven, Kapucijnenvoer 7, 3000 Leuven, Belgium; 2 Nuffield Department of Primary Care Health Sciences, University of Oxford, Woodstock Road, Oxford, OX26GG UK

**Keywords:** Antibiotics, Anti-bacterial agents, Prescriptions, Drug utilization, Pharmacy dispensing, Ambulatory care, Outpatient, Belgium

## Abstract

**Background:**

The desired effect of antibiotics is compromised by the rapid escalation of antimicrobial resistance. Children are particularly at high-risk for unnecessary antibiotic prescribing, which is owing to clinicians’ diagnostic uncertainty combined with parents’ concerns and expectations. Recent Belgian data on ambulatory antibiotic prescribing practices for children are currently lacking. Therefore, we aim to analyse different aspects of antibiotic prescriptions for children in ambulatory care.

**Methods:**

Pharmacy dispensing data on antibiotics for systematic use referring from 2010 to 2019 were retrieved from Farmanet, a database of pharmaceutical dispensations in community pharmacies. Population data were obtained from the Belgian statistical office (Statbel). Descriptive statistics were performed in Microsoft Excel. The Mann-Kendall test for trend analysis and the seasplot function for seasonality testing were conducted in R.

**Results:**

The past decade, paediatric antibiotic use and expenditures have relatively decreased in Belgian ambulatory care with 35.5% and 44.3%, respectively. The highest volumes of antibiotics for children are prescribed by GPs working in Walloon region and rural areas, to younger children, and during winter. The most prescribed class of antibiotics for children are the penicillins and the biggest relative reduction in number of packages is seen for the sulfonamides and trimethoprim and quinolone antibacterials.

**Conclusions:**

Paediatric antibiotic use has decreased in Belgian ambulatory care. Further initiatives are needed to promote prudent antibiotic prescribing in ambulatory care.

**Supplementary Information:**

The online version contains supplementary material available at 10.1186/s12887-021-03047-7.

## Background

Antibiotics are one of the most cost-effective, live-saving treatments and they may contribute to an extended lifespan. However, their effect is compromised by the rapid escalation of antimicrobial resistance (AMR) which is considered as a major global health threat [1]. Drug-resistant infections lead to approximately 700,000 deaths per year globally. This is expected to increase to ten million by 2050, with associated costs estimated as high as 100 trillion dollars worldwide if no action is taken [1].

Children are particularly at high-risk for unnecessary antibiotic prescribing [2, 3]. Acute infection or feverish illness is the most common reason for children to attend ambulatory care [4]. Only 1% of those children attending primary care will be diagnosed with a serious infection [5]. Distinguishing serious from non-severe infections is difficult, especially in the early stages of the disease when signs and symptoms are unspecific [5]. Furthermore, Belgian prescribing guidelines advise to restrict paediatric antibiotic use to certain indications under specific conditions [6, 7]. These guidelines are not always clear-cut, and still leave room for doubt and subjective assessment (e.g., “appearing seriously ill”, “less fluid intake”) [8]. Thus, physicians face diagnostic uncertainty, which can lead to inappropriate antibiotic prescribing, unnecessary referrals to hospital, and unwarranted additional testing due to concerns about a potential serious infection [9]. In addition, parents of acutely ill children might consult their general practitioner (GP) with specific concerns and expectations [2, 3, 10], which may result in the physician issuing a prescription for antibiotics to maintain a good doctor-patient relationship [11]. This enforces inappropriate antibiotic prescribing behaviour [1].

Belgian reports on antibiotic prescribing practices deduce that the largest volumes of antibiotics are consumed in the community [12]. These reports intermittently provide in-depth feedback to GPs on general prescription data [12, 13], but data for children alone is lacking. The National Institute for Health and Disability Insurance (NIHDI), RIZIV-INAMI, published the latest report concerning antibiotic use for children in Belgium [14]. However, this report is outdated (2012-2014) and does not provide sufficient details (e.g., no information about the specialty of the prescriber or the dispensing area).

Therefore, we aim to analyse Belgian antibiotic dispensing data on children in ambulatory care over a ten-year period regarding antibiotic subgroups, delivery month, specialty of the prescriber, age of the child, region, and rurality category.

## Methods

Pharmacy dispensing data on antibiotics for systematic use (Anatomical Therapeutic Chemical classification system ATC J01) referring from 2010 to 2019 were retrieved from Farmanet, a Belgian database containing data on pharmaceutical dispensations, delivered in community pharmacies and reimbursed by the compulsory health insurance [15]. We requested dispensing data for children from birth until 12 years of age, with age defined as the delivery year of the antibiotic minus the birth year of the entitled party. Dispensing data (i.e., the number of sold packages and NIHDI expenditures) were summarized by ATC level 3 (i.e., the therapeutic/pharmacological subgroup), delivery month, provider speciality (GP and specialist in paediatrics), age, region, and rurality type. Number of packages can be considered a good proxy for treatments [16]. Moreover, the Defined Daily Dose (DDD) is not a suitable metric for children as this is normally assigned based on use in adults [17]. Dose recommendations for children differ based on age and body weight [17]. Antibiotics for topical use (ATC D06A) and ophthalmologicals (ATC S01) were not considered.

Population data were obtained from the Belgian statistical office (Statbel) [18]. Belgium consists of three regions, namely Flanders (Northern part), Walloon region (Southern part), and Brussels Capital Region [19]. Further, we used the DEGURBA classification system of the European Commission [20] to label townships as “city”, “town/suburb”, or “rural”.

Descriptive statistics were performed in Microsoft Excel version 21.02. Statistical analysis was conducted with R version 4.0.3. Because of non-linearity, the nonparametric Mann-Kendall test was used for trend analysis. A *p*-value ≤ 0.05 was considered significant. The function seasplot was used to construct seasonal plots of time series objects which were created through the ts function.

## Results

The results below describe the number of packages of antibiotics for systemic use (ATC J01) delivered in Belgian public pharmacies to children aged zero to 12 years and associated NIHDI expenditures, calculated per 1000 inhabitants. Extensive data tables are included in Additional file 1.

### General trends from 2010 until 2019

**Fig. 1:**
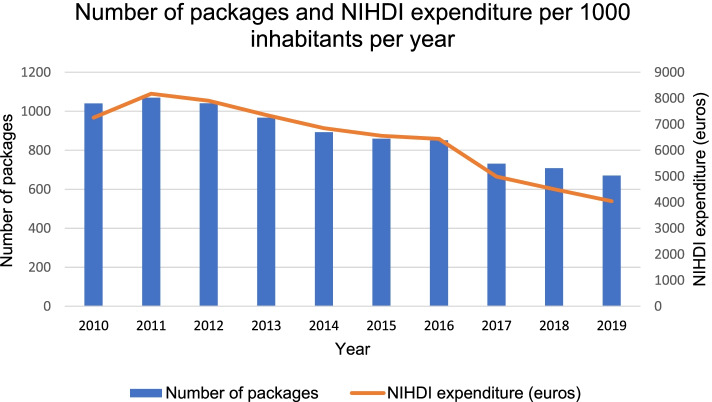
Number of packages and NIHDI expenditure per 1000 inhabitants per year (2010-2019). NIHDI: National Institute for Health and Disability Insurance

In 2019, 670 packages of antibiotics per 1000 inhabitants were delivered in Belgian public pharmacies to children. In the last decade, the number of packages of antibiotics and NIHDI expenditures per 1000 inhabitants have significantly decreased with relatively 35.5% (*p* < 0.001) and 44.3% (*p* < 0.001), respectively (Fig. 1). In absolute terms, this is a reduction of half a million of packages and 4.7 million euros. Between 2010 and 2011, the number of packages and NIHDI expenditures increased. From 2012, these numbers have only decreased, with the strongest fall in 2017. From 2015 onwards, NIHDI expenditures have been declining more sharply than the number of packages.

### ATC level 3

**Fig. 2:**
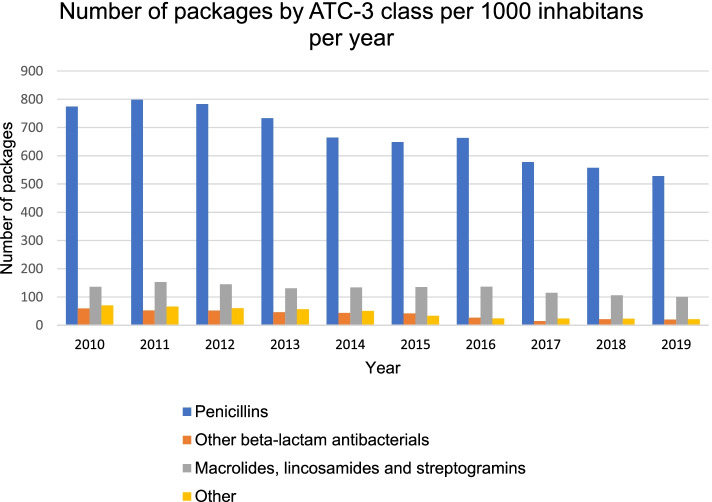
Number of packages by ATC-3 class per 1000 inhabitants per year (2010-2019). ATC: Anatomical Therapeutic Chemical classification system

The most prescribed classes of antibiotics for children are the penicillins (J01C, 78.8% of total antibiotic use for children in 2019) and the macrolides, lincosamides, and streptogramins (J01F, 14.9% in 2019) (Fig. 2). The biggest relative reduction in number of packages is seen for the sulfonamides and trimethoprim (J01E, -95.3%, *p* < 0.001), quinolone antibacterials (J01M, -68.2%, *p* = 0.002), beta-lactam antibacterials other than penicillin (J01D, -66.0%, *p* < 0.001), and amphenicols (J01B, -59.7%, *p* < 0.001) (Fig. 2).

### Months of the year

**Fig. 3:**
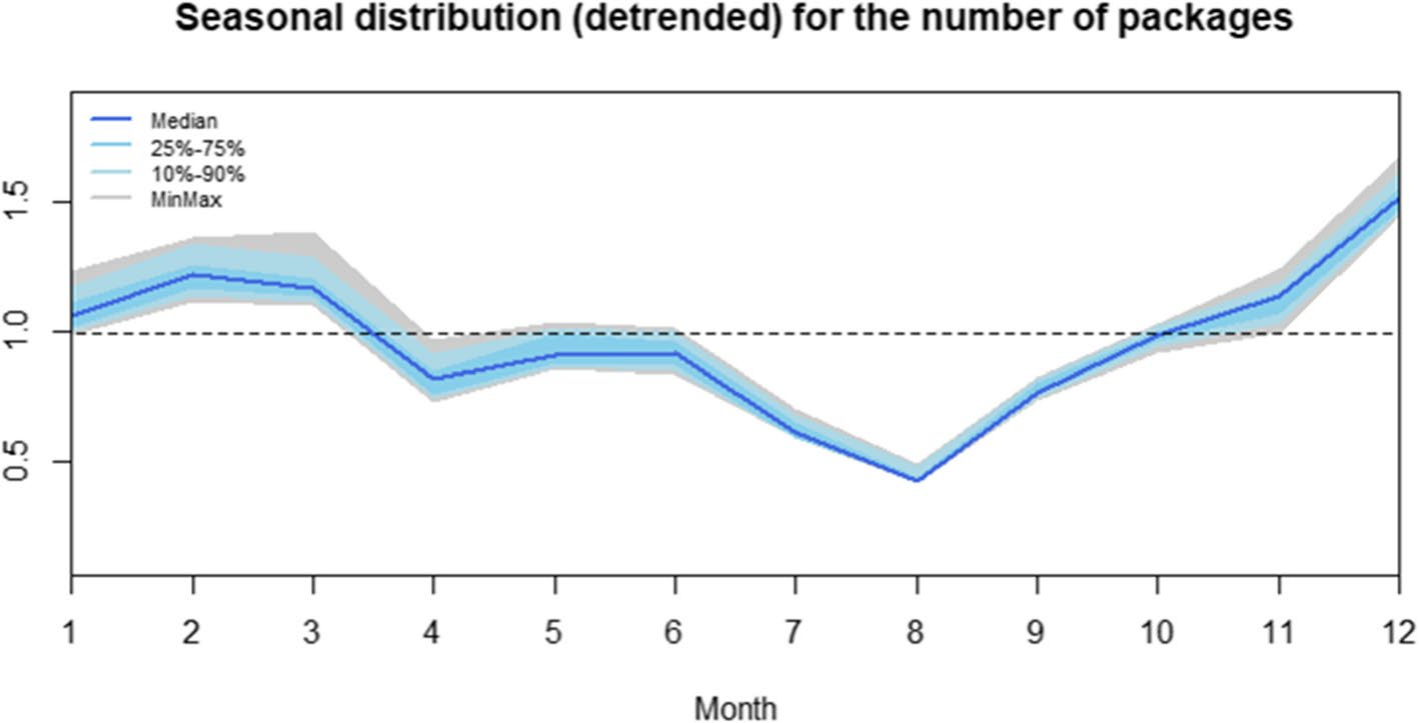
Seasonal distribution for the number of packages for every year between 2010 and 2019

In 2019, most antibiotics were delivered in February and December and these months have the highest NIHDI expenditure (Supplemental Fig. 1). The least antibiotics were delivered during summer, and especially in August, which also has the lowest NIHDI expenditure. There is evidence of seasonality (Fig. 3).

### Specialty

**Fig. 4:**
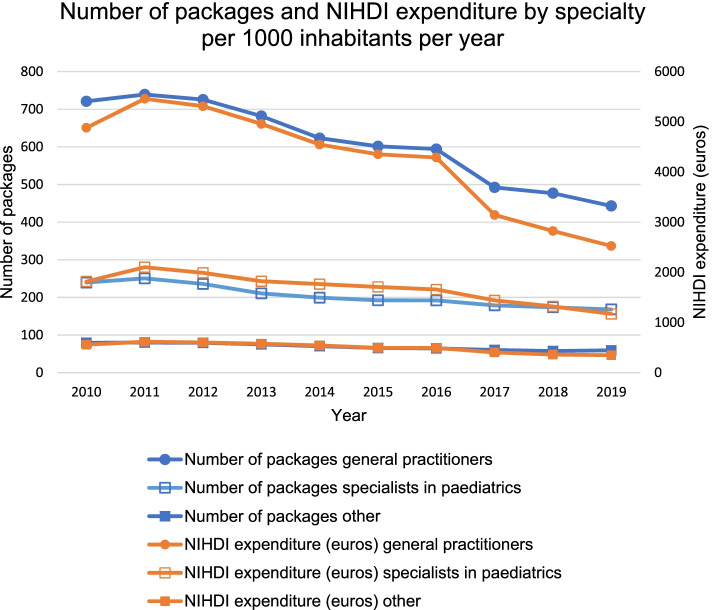
Number of packages and NIHDI expenditure by specialty per 1000 inhabitants per year (2010-2019). NIHDI: National Institute for Health and Disability Insurance

During the last ten years, the number of packages of antibiotics prescribed for children and NIHDI expenditures have significantly decreased for all specialties (Fig. 4). The number of packages has relatively declined with 38.6% for GPs (*p* < 0.001), with 29.8% (*p* < 0.001) for paediatricians, and with 25.4% (*p* < 0.001) for other medical doctors (e.g., dentists and pulmonologists). NIHDI expenditures dropped with 48.3% (*p* < 0.001) for GPs, with 35.60% (*p* < 0.001) for paediatricians, and with 37.5% (*p* < 0.001) for other medical doctors.

**Fig. 5:**
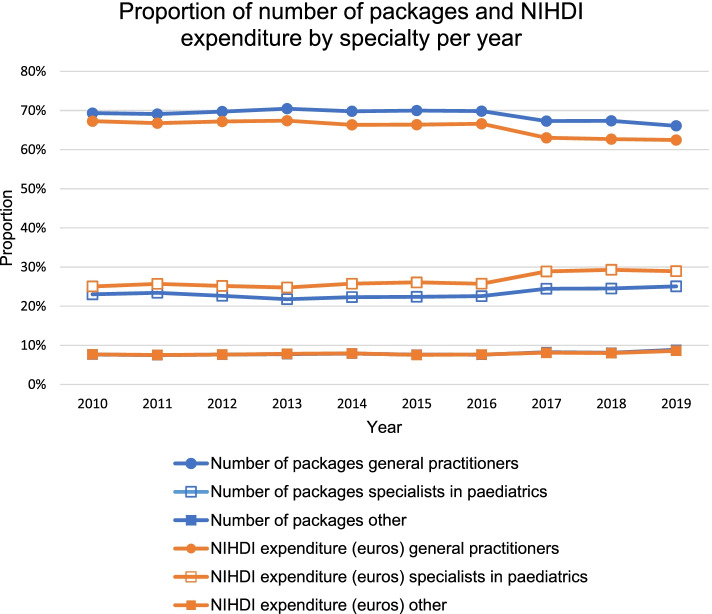
Proportion of number of packages and NIHDI expenditure by specialty per year (2010-2019). The graph for number of packages for the category ‘other’ is covered behind its graph for NIHDI expenditure. NIHDI: National Institute for Health and Disability Insurance

In 2019, 66.1% of antibiotics for children was prescribed by a GP and this accounted for 62.5% of NIHDI expenditures. Furthermore, 25.1% was prescribed by a specialist in paediatrics with associated NIHDI expenditures of 28.9%. Consequently, 8.8% of antibiotics for children was prescribed neither by a GP nor by a paediatrician, corresponding to a NIHDI expenditure of 8.6% (Fig. 5). Only the proportion of number of packages prescribed by other medical doctors increased significantly (RR +15.8%, *p* = 0.049), as compared to the number of packages prescribed by a GP (RR -4.7%, *p* = 0.371), or a paediatrician (RR +8.9%, *p* = 0.152) (Fig. 5). Nevertheless, the proportion of NIHDI expenditures had a significant trend for every specialty. For GPs, this has decreased (RR -7.2%, *p* = 0.007), while this has increased for paediatricians (RR +15.5%, *p* = 0.007) and other medical doctors (RR +12.1%, *p* = 0.032) (Fig. 5). In terms of absolute change, the number of packages prescribed by a GP has decreased with 3.3% (AR), while this has increased with 2.1% and 1.2% for paediatricians and other medical doctors, respectively. Likewise, the proportion of NIHDI expenditures for GPs has decreased with 4.8%, while this increased with 3.9% and 0.9% for paediatricians and other medical doctors, respectively.

### Age

**Fig. 6:**
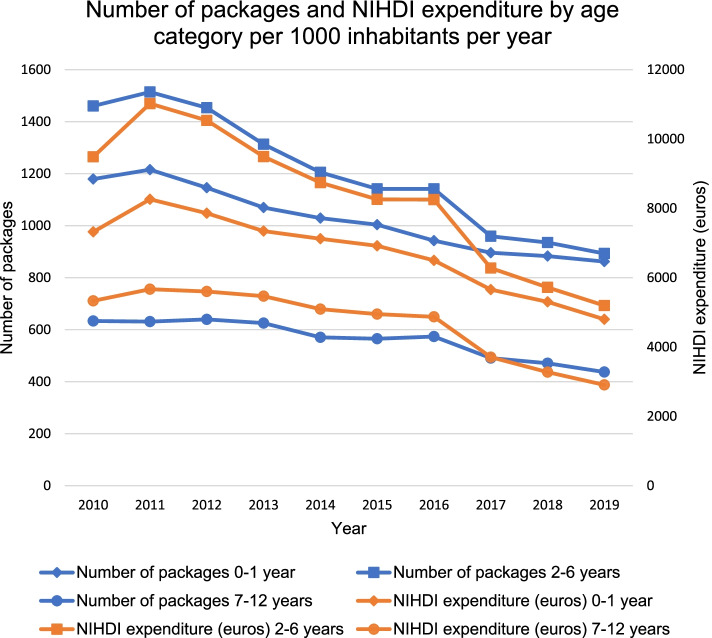
Number of packages and NIHDI expenditure by age category per 1000 inhabitants per year (2010-2019). NIHDI: National Institute for Health and Disability Insurance

In 2019, 18.2% of the antibiotic use in children was represented by age group of zero- to one-year-olds, 50.6% by two- to six-year-olds, and 31.2% by seven- to 12-year-olds. Considering the number of Belgian inhabitants per age category, we can conclude that more antibiotics are prescribed in children up to 6 years, compared to seven- to 12-year-olds (Fig. 6). Between 2010 and 2019, antibiotic use has relatively decreased with 26.9% (*p* < 0.001) for zero- to one-year-olds, with 38.9% (*p* < 0.001) for two- to six-year-olds, and with 31.0% (*p* = 0.001) for seven- to 12-year-olds (Fig. 6). NIHDI expenditures have decreased with 34.4% (*p* < 0.001), 45.2% (*p* < 0.001), and 45.5% (*p* < 0.001), respectively.

### Area

Antibiotic use has decreased for every region and rurality category in Belgium and is the lowest in Brussels Capital Region and cities and the highest in Walloon region and rural areas (Supplemental Figs. 2 and 3).

## Discussion

### Main findings and interpretation

In the last decade, the number of packages of antibiotics for systemic use delivered in public pharmacies to children and associated NIHDI expenditures have decreased. New antibiotic prescribing guidelines [21], several national antibiotic campaigns [16], and some local initiatives such as communication skills training [22] and discussing antibiotic prescription behaviour at local quality circles [23], are associated with significantly declining antibiotic use for children in Belgium. The change of the reimbursement conditions in 2017 [24] and 2018 [25] and making it mandatory for the pharmacist to deliver the cheapest antibiotic [26] have contributed to reducing NIHDI expenditures. Additionally, the difficulties associated with developing new antibiotics [27], together with the expiration of patents of agents that did develop successfully, promotes that the cheaper, generic antibiotics represent a bigger portion of the market [28]. As a consequence, the price of the original specialities must lower to compete with the generics [29], suppressing NIHDI expenditures. An overall decreasing trend in antibiotic use and expenditures has been observed for other high-income countries, such as France [30], Italy [31], Germany [32], and the Netherlands [32].

Similar to other high-income countries [30, 31, 33, 34], penicillins are in general the most commonly prescribed antibiotics for children. Alternations in prescribing guidelines, which is often a consequence of antimicrobial resistance and/or side effects [35], drug shortages, and the rising co-payment of the patient for certain drug classes [25] presumably result in shifts in use of the different antibiotic classes.

We found that most antibiotics are delivered during winter and the least during summer. During winter, there is a higher incidence of respiratory infections, which are mainly caused by viruses [16]. These viral respiratory tract infections may lead to a secondary bacterial infection such as pneumonia or otitis media where an antibiotic is often justified [2]. An Italian study observed the same seasonality pattern of prescriptions, which was shown to be in parallel with the trend of flu syndromes [31].

Although declining in the past ten years, most antibiotics for children are still prescribed by GPs, followed by paediatricians. In the United States, it was also observed that family practitioners account for the highest number of outpatient antibiotic prescriptions, followed by paediatricians, internists, and dentists [34]. However, they did not consider prescriptions for children separately. For paediatricians, the contribution of NIHDI expenditures is higher than for the number of packages. This implies that specialists in paediatrics prescribe more expensive drugs. Some children presenting to paediatricians require more specialistic, so more expensive, care, as they suffer from conditions such as asthma, leukaemia, immune deficiency, cystic fibrosis, and recurring urinary tract infections [36]. Moreover, expensive, and often more toxic, alternative antibiotics must be used when pathogens become resistant to first-line therapy [37]. We also found a significant increase in cost and number of antibiotic packages prescribed by specialists other than GPs or paediatricians (e.g., ear, nose, and throat physicians, pulmonologists, dermatologists, internal medicine specialists, and dentists) [12]. Therefore, we hypothesize that antibiotic campaigns were less effective for these doctors. Besides, extensive antibiotic guidelines are less readily available for these doctors, compared to primary care physicians.

Although decreasing for all age categories, still more antibiotics are prescribed for younger children. This is as expected since younger children are more at risk of serious infections, requiring more cautious management [38]. This is also observed in Italy [31, 33], Denmark [33], France [30], and the United States [34].

As with our study, geographic variations in antibiotic consumption have also been observed for Italy [31] and the United States [34].

### Strengths and limitations

This is an in-depth analysis of recent antibiotic dispensing data for children in Belgium. Farmanet is a reliable source of antibiotic prescription data, as opposed to self-reporting by patients. This database contains enough detail regarding the age and place of residence of the child and the specialty of the prescribing healthcare professional. We used the number of packages as a proxy of antibiotic use, as DDD is not a suitable metric for children [17].

Our research has some limitations as well. It is crucial to keep in mind that, given the data retrieved from Farmanet, exposure to antibiotics is underestimated because the practice of self-medication is unknown, i.e., the use of leftovers or drugs obtained from elsewhere (e.g., abroad, friends and family) [39]. In fact, this could represent an important source of inappropriate antimicrobial use [39]. Belgian antibiotic use is even more underestimated, as we did not consider the prescriptions of antibiotics for topical use and ophthalmologicals. These classes of antibiotics fall out of the scope of this research, namely diagnostic uncertainty in acutely ill children. On the other hand, it is impossible to estimate whether the patient took the prescribed drug and that it was taken as prescribed by the doctor (i.e., right dose, time of the day, and duration) [40].

Moreover, our conclusions should be interpreted with caution, as correction for population growth and/or decline is imprecise. Farmanet calculates age as delivery year of the antibiotic minus birth year, overestimating the actual age of the children, while Statbel contains data for the first of January of each year. This means that data retrieved from Farmanet and Statbel cannot be perfectly aligned to perform trend analysis per 1000 inhabitants. However, correcting for alternations in the population allows generalisability to other settings.

### Implications for research

This research is a critical step in identifying where antibiotic prescribing practices can be improved. Our data suggest that physicians working in Walloon region and rural areas prescribe more antibiotics per 1000 inhabitants. Besides, in the past ten years, other medical doctors than GPs and paediatricians have proportionally prescribed more antibiotics for children.

The lack of information about the indication the drug was prescribed for, the dosing frequency, and the duration of therapy, make it impossible to estimate whether the use of antibiotics was appropriate based on these data. Further research is needed to investigate the appropriateness of prescriptions.

Moreover, further research should investigate temporal trends of broad and narrow spectrum antibiotic use, as we did not provide data on the individual antibiotics. Physicians prescribe broad-spectrum antibiotics because of diagnostic uncertainty, stimulating antimicrobial resistance [41]. This diagnostic uncertainty occurs for instance when the physician does not know whether the illness is of viral or bacterial origin or, when considered bacterial, which bacteria is the causative micro-organism.

Future studies should aim to analyse whether COVID-19 has had an impact on antibiotic use in Belgium. We hypothesize that, similarly to England [42], the number of prescriptions will have declined, but when compared to the number of doctor’s appointments, this number is higher than expected. During COVID-19, there is a higher rate of remote/telephone consultations, leading to higher diagnostic uncertainty by which physicians tend to prescribe an antibiotic more easily [42].

## Conclusions

The past decade, paediatric antibiotic use and expenditures have decreased in Belgian ambulatory care. The highest volumes of antibiotics for children are prescribed by GPs working in Walloon region and rural areas, to younger children, and during winter. Prudent antibiotic prescribing should be continuously promoted in ambulatory care.

## Supplementary Information

**Additional file 1.** Contains simplified tables of the requested pharmacy dispensing data, corrected for the number of inhabitants. This can be found online.

**Additional file 2: Supplemental figure 1**. Number of packages and NIHDI expenditure per 1000 inhabitants by month (2019). **Supplemental figure 2**. Number of packages (A) NIHDI expenditure (B) by region per 1000 inhabitants per year (2010-2019). **Supplemental figure 3**. Number of packages (A) and NIHDI expenditure (B) by rurality category per 1000 inhabitants per year (2010-2019).

## Data Availability

All data analysed during this study are included in Additional file 1.

## Abbreviations

AMR: Antimicrobial resistance
AR: Absolute risk
ATC: Anatomical Therapeutic Chemical classification system
DDD: Defined Daily Dose
GP: General practitioner
NIHDI: National Institute for Health and Disability Insurance
RR: Relative risk
Statbel: The Belgian statistical office

## References

[1] Machowska A, Stalsby LC. Drivers of Irrational Use of Antibiotics in Europe. Int J Environ Res Public Health. 2018;16(1):1–14. 10.3390/ijerph16010027 PMC6338985 30583571

[2] Ashdown HF, Raisanen U, Wang K, Ziebland S, Harnden A, investigators A (2016). Prescribing antibiotics to 'at-risk' children with influenza-like illness in primary care: qualitative study. BMJ Open.

[3] de Bont EG, van Loo IH, Dukers-Muijrers NH, Hoebe CJ, Bruggeman CA, Dinant GJ (2013). Oral and topical antibiotic prescriptions for children in general practice. Arch Dis Child.

[4] Verbakel JY, Lemiengre MB, De Burghgraeve T, De Sutter A, Bullens DM, Aertgeerts B (2014). Diagnosing serious infections in acutely ill children in ambulatory care (ERNIE 2 study protocol, part A): diagnostic accuracy of a clinical decision tree and added value of a point-of-care C-reactive protein test and oxygen saturation. BMC Pediatr.

[5] Van den Bruel A, Thompson M (2014). Research into practice: acutely ill children. Br J Gen Pract.

[6] RIZIV (2016). Het Rationeel Gebruik van de Antibiotica bij het Kind in de Ambulante Zorg.

[7] BAPCOC. Belgische Gids voor Anti-Infectieuze Behandeling in de Ambulante Praktijk 2019 [Available from: https://www.bcfi.be/nl/chapters/12?frag=8000010. Accessed 20/10/2020.]

[8] Lemiengre MB, Verbakel JY, Colman R, Van Roy K, De Burghgraeve T, Buntinx F (2018). Point-of-care CRP matters: normal CRP levels reduce immediate antibiotic prescribing for acutely ill children in primary care: a cluster randomized controlled trial. Scand J Prim Health Care.

[9] Verbakel JY, Lee JJ, Goyder C, Tan PS, Ananthakumar T, Turner PJ (2019). Impact of point-of-care C reactive protein in ambulatory care: a systematic review and meta-analysis. BMJ Open.

[10] Lucas PJ, Cabral C, Hay AD, Horwood J (2015). A systematic review of parent and clinician views and perceptions that influence prescribing decisions in relation to acute childhood infections in primary care. Scand J Prim Health Care.

[11] Lemiengre MB, Verbakel JY, Colman R, De Burghgraeve T, Buntinx F, Aertgeerts B (2018). Reducing inappropriate antibiotic prescribing for children in primary care: a cluster randomised controlled trial of two interventions. Br J Gen Pract.

[12] Leroy R, Christiaens W, Maertens de Noordhout C, Hanquet G. Proposals for a more effective antibiotic policy in Belgium - Report. KCE. 2019.

[13] ECDC (2019). Antimicrobial consumption in the EU/EEA.

[14] RIZIV (2016). Infospot - Antibiotica bij het kind in de ambulante zorg.

[15] RIZIV. Statistieken over geneesmiddelen afgeleverd in openbare apotheken (Farmanet) 2013 [Available from: https://www.riziv.fgov.be/nl/statistieken/geneesmiddel/Paginas/Statistieken-geneesmiddelen-apotheken-farmanet.aspx. Accessed 11/12/2020.]

[16] Bruyndonckx R, Coenen S, Hens N, Vandael E, Catry B, Goossens H (2020). Antibiotic use and resistance in Belgium: the impact of two decades of multi-faceted campaigning. Acta Clin Belg.

[17] World Health Organisation. Defined Daily Dose (DDD) 2020 [Available from: https://www.who.int/toolkits/atc-ddd-toolkit/about-ddd. Accessed 8/12/2020.]

[18] STATBEL. Structuur van de bevolking 2020 [Available from: https://statbel.fgov.be/nl/themas/bevolking/structuur-van-de-bevolking#news. Accessed 24/12/2020.]

[19] Vlaams Parlement. Structuur van België 2021 [Available from: https://www.vlaamsparlement.be/over-het-vlaams-parlement/het-vlaams-parlement-het-politieke-landschap/structuur-van-belgi%C3%AB. Accessed 26/05/2021.]

[20] European Commission. Degree of Urbanisation (DEGURBA) - Local Administrative Units 2019 [Available from: https://ec.europa.eu/eurostat/ramon/miscellaneous/index.cfm? TargetUrl=DSP_DEGURBA. Accessed 11/12/2020.]

[21] Chevalier P, Leconte S, De Sutter A. Belgische Gids voor Anti-Infectieuze Behandeling in de Ambulante Praktijk. BAPCOC. 2012;1:1–84.

[22] E-learning om antibioticaverbruik te verminderen (2017). Domus Medica.

[23] RIZIV. De LOK, lokale kwaliteitsgroep 2014 [Available from: https://www.inami.fgov.be/nl/professionals/individuelezorgverleners/artsen/kwaliteit/accreditering/Paginas/artsen-accreditering-LOK-index.aspx. Accessed 18/11/2020.]

[24] RIZIV. Antibiotica : terugbetaling vanaf 1 mei 2017 2017 [Available from: https://www.riziv.fgov.be/nl/themas/kost-terugbetaling/door-ziekenfonds/geneesmiddel-gezondheidsproduct/terugbetalen/specialiteiten/wijzigingen/Paginas/antibiotica-20170501.aspx. Accessed 26/10/2020.]

[25] RIZIV. Antibiotica die tot de klasse van de (fluoro) chinolonen behoren: terugbetaling vanaf 1 mei 2018 2018 [Available from: https://www.riziv.fgov.be/nl/themas/kost-terugbetaling/door-ziekenfonds/geneesmiddel-gezondheidsproduct/terugbetalen/specialiteiten/wijzigingen/Paginas/antibiotica-fluoro-chinolonen.aspx. Accessed 26/10/2020.]

[26] RIZIV (2012). Afleveren van het goedkoopste geneesmiddel bij - voorschrijven op stofnaam - antibiotica en antimycotica.

[27] Bosley H, Henshall C, Appleton JV, Jackson D (2018). A systematic review to explore influences on parental attitudes towards antibiotic prescribing in children. J Clin Nurs.

[28] Europese Raad. EU neemt maatregelen aan om producenten van generieke geneesmiddelen te ondersteunen 2019 [Available from: https://www.consilium.europa.eu/nl/press/press-releases/2019/05/14/eu-adopts-measures-in-support-of-generic-pharmaceuticals-producers/. Accessed 23/03/2021.]

[29] FOD Economie. Generieke geneesmiddelen 2019 [Available from: https://economie.fgov.be/nl/themas/verkoop/prijsbeleid/gereguleerde-prijzen/geneesmiddelen-voor-menselijk/generieke-geneesmiddelen. Accessed 28/01/2021.]

[30] Trinh NTH, Bruckner TA, Lemaitre M, Chauvin F, Levy C, Chahwakilian P, et al. Association between National Treatment Guidelines for Upper Respiratory Tract Infections and Outpatient Pediatric Antibiotic Use in France: An Interrupted Time-Series Analysis. J Pediatr. 2020;216:88–94. 10.1016/j.jpeds.2019.09.017 31610933

[31] Cangini AA-O, Fortinguerra FA-O, Di Filippo A, Pierantozzi A, Da Cas R, Villa F (2020). Monitoring the community use of antibiotics in Italy within the National Action Plan on antimicrobial resistance. Br Pharmacol Soc.

[32] Gradl GA-OX, Teichert MA-O, Kieble M, Werning J, Schulz MA-O. Comparing outpatient oral antibiotic use in Germany and the Netherlands from 2012 to 2016. Pharmacoepidemiol Drug Saf. 2018(27):1344–55. 10.1002/pds.4643 PMC6585743 30264894

[33] Lusini G, Lapi F, Fau-Sara B, Sara B Fau-Vannacci A, Vannacci A Fau-Mugelli A, Mugelli A Fau-Kragstrup J, Kragstrup J Fau-Bjerrum L (2009). Antibiotic prescribing in paediatric populations: a comparison between Viareggio, Italy and Funen, Denmark. Eur J Pub Health.

[34] Hicks LA, Bartoces MG, Roberts RM, Suda KJ, Hunkler RJ, Taylor TH Jr, et al. US outpatient antibiotic prescribing variation according to geography, patient population, and provider specialty in 2011. CID. 2015;60:1308–16. 10.1093/cid/civ076 25747410

[35] BCFI. Infecties [Available from: https://www.bcfi.be/nl/chapters/12?frag=9423. Accessed 16/12/2020.]

[36] UZ Leuven. Kindergeneeskunde 2021 [Available from: https://www.uzleuven.be/nl/kindergeneeskunde. Accessed 28/01/2021.]

[37] Ventola CL (2015). The antibiotic resistance crisis: part 1: causes and threats. P T.

[38] Elshout G, van Ierland Y, Bohnen AM, de Wilde M, Oostenbrink R, Moll HA (2013). Alarm signs and antibiotic prescription in febrile children in primary care: an observational cohort study. Br J Gen Pract.

[39] Rossignoli A, Clavenna A, Bonati M (2007). Antibiotic prescription and prevalence rate in the outpatient paediatric population: analysis of surveys published during 2000-2005. Eur J Clin Pharmacol.

[40] Clavenna A, Bonati M (2009). Drug prescriptions to outpatient children: a review of the literature. Eur J Clin Pharmacol.

[41] ECDC. Facts about antimicrobial resistance 2021 [Available from: https://www.ecdc.europa.eu/en/antimicrobial-resistance/facts. Accessed 27/01/2021.]

[42] Armitage R, Nellums LB. Antibiotic prescribing in general practice during COVID-19. Lancet Infect Dis. 2020;21:e144. 10.1016/S1473-3099(20)30917-8 PMC9761101 33275941

