# Correction to: Using Belgian pharmacy dispensing data to assess antibiotic use for children in ambulatory care

**DOI:** 10.1186/s12887-022-03152-1

**Published:** 2022-03-11

**Authors:** Hannelore Dillen, Ruben Burvenich, Tine De Burghgraeve, Jan Y. Verbakel

**Affiliations:** 1 EPI-Centre, Department of Public Health and Primary Care, KU Leuven, Kapucijnenvoer 7, 3000 Leuven, Belgium; 2 Nuffield Department of Primary Care Health Sciences, University of Oxford, Woodstock Road, Oxford, OX26GG UK

**Correction to: BMC Pediatr 22, 12 (2022)**

**https://doi.org/10.1186/s12887-021-03047-7**

Following the publication of the original article [1], the authors identified the following errors:

1. The legends of figures 4 and 5 should be:

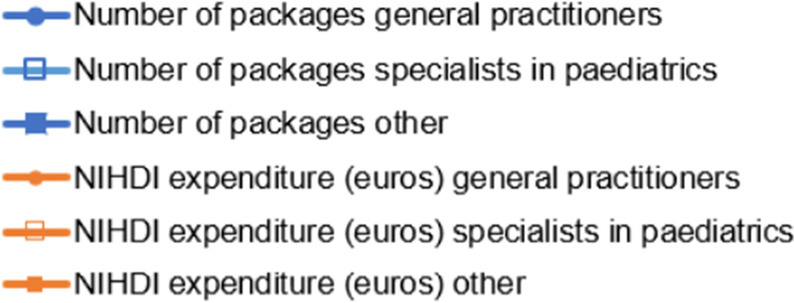

2. Additional file 2: Updated file captured as supplementary file of this paper.

## Supplementary Information

**Additional file 2**: **Supplemental figure 1**. Number of packages and NIHDI expenditure per 1000 inhabitants by month (2019). **Supplemental figure 2**. Number of packages (A) NIHDI expenditure (B) by region per 1000 inhabitants per year (2010-2019). **Supplemental figure 3**. Number of packages (A) and NIHDI expenditure (B) by rurality category per 1000 inhabitants per year (2010-2019).
