# Supplementary material for: Using Belgian pharmacy dispensing data to assess antibiotic use for children in ambulatory care
Source: BMC Pediatr. 2022 Jan 3;22:12. doi: 10.1186/s12887-021-03047-7 (PMC8720940; doi:10.1186/s12887-021-03047-7)
Supplement: Supplementary file 2 — Additional file 2: Supplemental figure 1. Number of packages and NIHDI expenditure per 1000 inhabitants by month (2019). Supplemental figure 2. Number of packages (A) NIHDI expenditure (B) by region per 1000 inhabitants per year (2010-2019). Supplemental figure 3. Number of packages (A) and NIHDI expenditure (B) by rurality category per 1000 inhabitants per year (2010-2019). [file 12887_2021_3047_MOESM2_ESM.docx]

**Supplemental figure 1. Number of packages and NIHDI expenditure per 1000 inhabitants by month (2019).**

NIHDI: National Institute for Health and Disability Insurance.

**Supplemental figure 2. Number of packages (A) NIHDI expenditure (B) by region per 1000 inhabitants per year (2010-2019).**

NIHDI: National Institute for Health and Disability Insurance.

**Supplemental figure 3. Number of packages (A) and NIHDI expenditure (B) by rurality category per 1000 inhabitants per year (2010-2019).**

NIHDI: National Institute for Health and Disability Insurance.
